# Effects of biochar, zeolite and mycorrhiza inoculation on soil properties, heavy metal availability and cowpea growth in a multi-contaminated soil

**DOI:** 10.1038/s41598-023-33712-z

**Published:** 2023-04-24

**Authors:** Ehab A. Ibrahim, Mohamed A. A. El-Sherbini, El-Metwally M. Selim

**Affiliations:** 1 Vegetables Research Department, Horticulture Research Institute, Agricultural Research Center, 9 Cairo University St., Orman, Giza, Egypt; 2 Department of Soil Sciences, Faculty of Agriculture, Damietta University, Damietta, 34517 Egypt

**Keywords:** Environmental sciences, Environmental chemistry, Pollution remediation

## Abstract

Heavy metal pollution of agricultural soil has become a major serious concern. The development of suitable control and remediation strategies for heavy metal contaminated soil has become critical. The outdoor pot experiment was conducted to investigate the effect of biochar, zeolite, and mycorrhiza on the bioavailability reduction of heavy metals and its subsequent effects on soil properties and bioaccumulation in plants as well as the growth of cowpea grown in highly polluted soil. Zeolite, biochar, mycorrhiza, zeolite with mycorrhiza, biochar with mycorrhiza, and soil without any modifications were the six treatments used. The experiment was conducted with a completely randomized design and four replications. The results indicated that the combination of biochar with mycorrhiza had the highest values of root and shoot dry weight and the lowest heavy metal concentrations in root and shoot as well as bioconcentration and translocation factors for all heavy metals. The highest significant reductions in the availability of heavy metals over the control were found with biochar with mycorrhiza*,* which were 59.1%, 44.3%, 38.0%, 69.7%, 77.8%, 77.2% and 73.6% for Cd, Co, Cr, Cu, Ni, Pb and Zn, respectively. The application of biochar and zeolite either alone or in combination with mycorrhiza increased significantly soil pH and EC compared to mycorrhiza treatment and untreated soil. It can be concluded that the combination of biochar and mycorrhizal inoculation has great potential as a cost-effective and environmentally technique for enhancing heavy metal immobilization, lowering heavy metal availability and plant uptake, and improving cowpea plant growth.

## Introduction

Heavy metal pollution of agricultural soils has been recorded in many regions throughout the world. The main sources of heavy metals in agricultural soil are the excessive application of sewage sludge, agrochemicals products, wastewater and organic manure^1^. Heavy metal accumulation was higher in vegetable soils than in other agricultural soils^2^. These metals have detrimental consequences on soil fertility, plant development and yield, and food safety. Therefore, finding effective remediation approaches for lowering heavy metal bioavailability in the soil resulting in lower plant uptake and phytotoxicity of heavy metals, which led to improved plant growth is critical. The safe and cost-efficient approach to remediate heavy metals in soil is the use of stabilizing agents. Some soil amendments such as biochar and zeolite and its effect on heavy metals have been identified as cost-effective methods for the immobilization of toxic metals^3,4^^.^

Biochar is a carbon-rich substance produced from organic feedstocks at high temperatures (> 300 °C) under oxygen-limited conditions. Biochar is a very porous substance with a large specific surface area^5^. It's been proven that biochar improves soil fertility and properties as well as helps to stabilize heavy metals^6^, thereby decreasing the phytoavailability of heavy metals through electrostatic attraction, physical adsorption, ion exchange, co-precipitation, and complexation^7^. Moreover, biochar enhances nitrogen fixation by improving the symbiotic performance of legumes with rhizobia^8^. Zeolites are crystalline and alkaline earth metals or hydrated aluminosilicate alkali metals with negatively charged positions and high specific surface area that can efficiently remove metals^9^. Zeolites have a great potential for remediation of heavy metal-contaminated soil by producing holes for water retention and heavy metal adsorption^10^, and due to their high cation-exchange capacity and hence capability to absorb heavy metals and so limit their phytoavailability^11^.

The use of large-surface-area amendments in heavy metal-contaminated soil improves plant growth, which can be linked to these components' involvement in lowering heavy metal availability in the soil and, as a result, encouraging soil microbial activity^12^. Mycorrhiza plays a vital role in plant growth by enabling nutrient gain from soils. Furthermore, mycorrhiza plays a significant role in the remediation of contaminated soils. The inoculation of mycorrhiza in contaminated soils reduces the negative effects of heavy metals. It has been shown to be able to survive toxic metals and to act as a filtration barrier to reduce heavy metals from the transformation in the soil–plant system, affecting the uptake and accumulation of heavy metals in plants and decreasing their toxicity^13^. Several studies have shown that arbuscular mycorrhizal fungi (AMF) reduces heavy metal toxicity in a variety of plants, and to maximize mycorrhiza's efficacy in toxic metal reduction, a variety of amendments have shown promising results in combination with mycorrhiza such as biochar^14^ and zeolite^15^.

Cowpea (*Vigna ungiculata* L.) is one of the legume crops valued for its high nutritional value, which provides protein, minerals, and vitamins for both humans and animals. It is an essential part of sustainable agriculture. It can fix nitrogen (N) from the atmosphere and grow well on infertile soils. However, cowpea is among the crops that readily absorb heavy metals, which can cause major health problems in humans^16^. Excessive accumulation of heavy metals in the soil results in a gradual decrease in plant growth, photosynthetic activity and production^17^**.** Many synthetic and natural amendments can decrease heavy metal extractability from contaminated soils. However, they are unable to increase microbial activity, soil productivity and plant growth. Soil treatment with mycorrhiza, biochar, and zeolite is considered an environmentally beneficial and cost-effective remediation technique. However, there is a scarcity of current study findings on the interaction of mycorrhiza with biochar or zeolite in soil remediation for vegetable production. Therefore, the objective of this study was to evaluate the effect of mycorrhiza and soil ameliorants (biochar and zeolite) and their mixture on soil characteristics, transformation and bioaccumulation of heavy metals in plants, and the availability of heavy metals in the soil as well as the growth of cowpea grown on heavily polluted soil.

## Results

### Plant dry biomass

**Figure 1:**
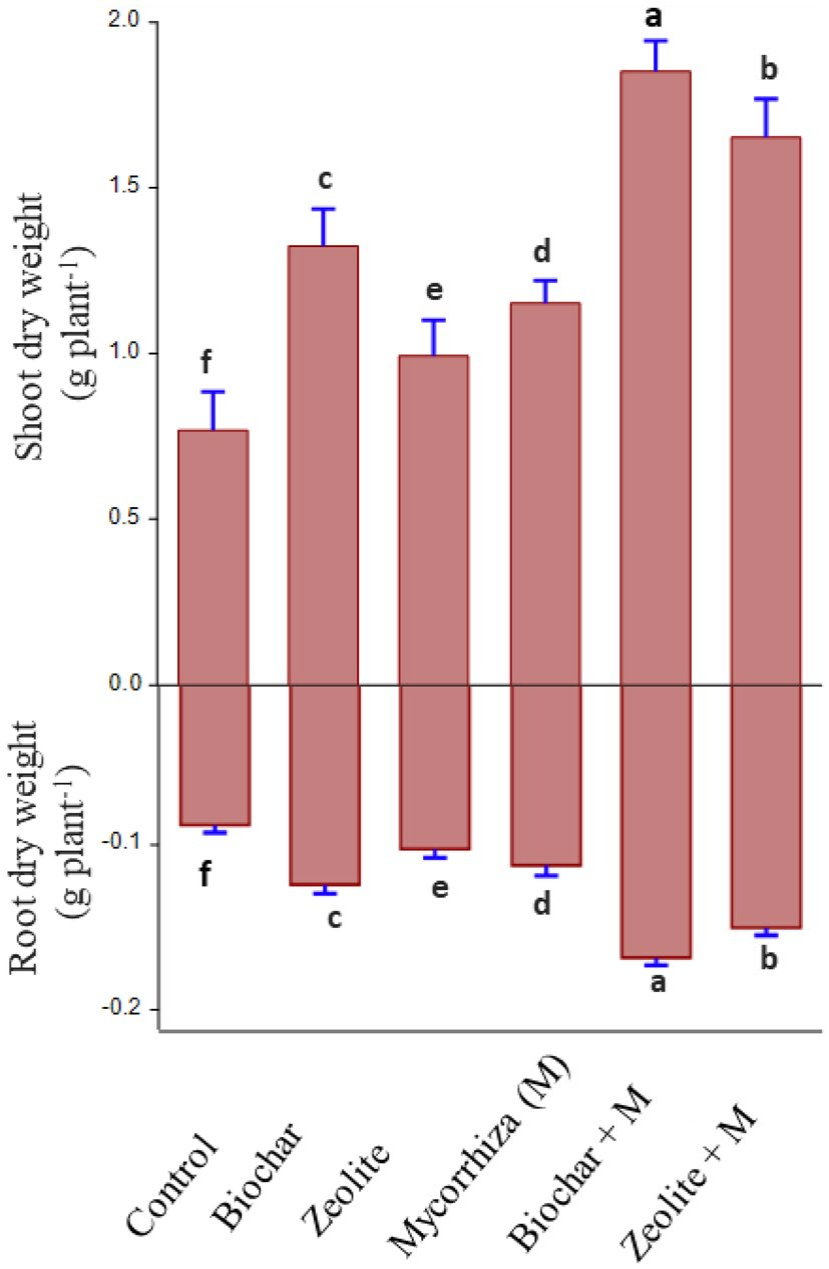
Effect of soil amendments and mycorrhiza on root and shoot dry weight of cowpea plant grown in contaminated soil. Error bars are the standard deviation. (Different letters above bars represent significant differences at *P* < 0.05 among various treatments. Bars with the same letters represent no significant difference).

The dry weight of the root and shoot was significantly increased with different treatments compared to the control (Fig. 1). The combination of biochar with mycorrhiza had the highest values followed by the combination of zeolite with mycorrhiza compared to other treatments. The treatment of biochar with mycorrhiza increased root and shoot dry weight by 92.6% and 141% respectively, compared to untreated soil.

### Heavy metal concentrations in cowpea plants

The changes in both root and shoot heavy metal concentrations of the seven heavy metals under different treatments are shown in Table 1. The highest concentrations were recorded for plants grown in untreated soil. In contrast, the lowest concentrations were found in the treatment of biochar followed by zeolite, especially in the presence of mycorrhiza. The treatment of biochar with mycorrhiza decreased concentrations of Cd, Co, Cr, Cu, Ni, Pb and Zn by 27.3%, 49.1%, 12.1%, 28.1%, 30.3%, 69.9% and 25.6% in roots, and by 43.6%, 74.8%, 22.6%, 32.2%, 37.3%, 82.1% and 29.7% in shoots, respectively, compared to untreated soil. The great decreases were found in the root and shoot Pb concentrations (69.9% and 82.1%, respectively).

**Table 1:** Effect of soil amendments and *mycorrhiza* on heavy metals concentration (mg kg^**-**1^ dw) in root and shoot tissues of cowpea plant grown in contaminated soil.

Treatments	Cd	Co	Cr	Cu	Ni	Pb	Zn
Root tissues							
Ziolite (Z)	4.25 b	13.25 b	169.46 b	53.78 b	45.14 b	16.11 b	210.46 ab
Biochar (B)	4.01 bc	11.33 c	160.63 d	47.17 d	39.28 d	11.09 d	188.66 cd
Mycorhiza (M)	4.31 b	13.10 b	164.30 c	50.67 c	41.16 c	13.15 c	200.36 bc
Z + M	3.73 cd	9.58 d	156.53 e	43.68 e	36.52 e	8.41 e	175.26 de
B + M	3.57 d	8.53 e	152.26 f.	41.57 f.	33.71 f.	5.85 f.	164.70 e
Control	4.91 a	14.75 a	173.20 a	57.79 a	48.39 a	19.41 a	221.43 a
LSD at 0.05	0.435	0.432	0.529	0.859	0.613	1.032	13.412
Shoot tissues							
Ziolite (Z)	2.76 a	9.55 b	88.32 b	48.83 b	36.59 b	9.22 b	148.26 b
Biochar (B)	2.77 a	6.34 d	80.52 d	41.89 d	30.90 d	5.30 d	130.70 d
Mycorhiza (M)	2.55 a	8.21 c	84.13 c	44.71 c	33.75 c	7.68 c	139.53 c
Z + M	2.00 b	4.28 e	75.54 e	38.75 e	27.84 e	3.33 e	121.60 e
B + M	1.64 b	2.68 f.	71.87 f.	35.57 f.	24.87 f.	2.04 f.	111.76 f.
Control	2.91 a	10.62 a	92.84 a	52.46 a	39.69 a	11.38 a	158.96 a
LSD at 0.05	0.458	0.424	0.394	0.510	0.448	0.797	1.940

Means followed with similar letters within the same column are not different significantly at *P* < 0.05 level of probability based on LSD test.

### Bioconcentration factor (BCF)

The BCF of the seven heavy metals under the different treatments of soil amendments and mycorrhiza are shown in Table 2. All treatments had significant effects on BCF compared to the untreated soil. The lowest effects were recorded with the application of biochar with mycorrhiza followed by the combination of zeolite with mycorrhiza for all heavy metals. The treatment of biochar with mycorrhiza shows that the BCF decreased up to 69.74%, 42.25%, 30.27%, 28.11%, 27.26%, 25.63% and 12.16% for Pb, Co, Ni, Cu, Cd, Zn and Cr, respectively.

**Table 2:** Effect of soil amendments and *mycorrhiza* on bioconcentration factor (BCF) of cowpea plants grown in contaminated soil.

Treatments	Cd	Co	Cr	Cu	Ni	Pb	Zn
Ziolite (Z)	0.766 b	0.527 b	0.853 b	0.808 b	0.620 b	0.162 b	0.950 b
Biochar (B)	0.721 bc	0.451 c	0.808 d	0.709 d	0.539 d	0.112 d	0.851 d
Mycorhiza (M)	0.776 b	0.521 b	0.827 c	0.761 c	0.565 c	0.132 c	0.904 c
Z + M	0.671 cd	0.381 d	0.788 e	0.656 e	0.501 e	0.085 e	0.791 e
B + M	0.643 d	0.339 e	0.766 f.	0.624 f.	0.463 f.	0.059 f.	0.743 f.
Control	0.884 a	0.587 a	0.872 a	0.868 a	0.664 a	0.195 a	0.999 a
LSD at 0.05	0.078	0.017	0.003	0.013	0.008	0.010	0.022

Means followed with similar letters within the same column are not different significantly at *P* < 0.05 level of probability based on LSD test.

### Translocation factor (TF)

The TF values for seven heavy metals were significantly reduced with all amendments compared to the control (Table 3). The least values of TF were detected after amending metal-contaminated soil with biochar in combination with mycorrhiza. This treatment decreased significantly the TF values of Cd, Co, Cr, Cu, Ni, Pb and Zn by 22.5%, 51.0%, 12.1%, 5.8%, 9.8%, 40.2% and 5.4%, respectively, over that in the control.

**Table 3:** Effect of soil amendments and *mycorrhiza* on translocation factor (TF) of cowpea plants grown in contaminated soil.

Treatments	Cd	Co	Cr	Cu	Ni	Pb	Zn
Ziolite (Z)	0.662 ab	0.636 a	0.521 b	0.909 a	0.814 a	0.577 a	0.704 ab
Biochar (B)	0.699 a	0.559 b	0.501 d	0.890 b	0.792 b	0.471 b	0.692 bc
Mycorhiza (M)	0.593 ab	0.627 a	0.512 c	0.883 b	0.824 a	0.583 a	0.696 b
Z + M	0.536 bc	0.447 c	0.482 e	0.889 b	0.766 c	0.397 c	0.694 bc
B + M	0.459 c	0.310 d	0.471 f.	0.856 c	0.742 d	0.352 c	0.678 bc
Control	0.592 ab	0.633 a	0.536 a	0.909 a	0.823 a	0.589 a	0.717 a
LSD at 0.05	0.132	0.034	0.001	0.016	0.012	0.059	0.017

Means followed with similar letters within the same column are not different significantly at *P* < 0.05 level of probability based on LSD test.

### The content of available heavy metals in the soil after harvest

**Figure 2:**
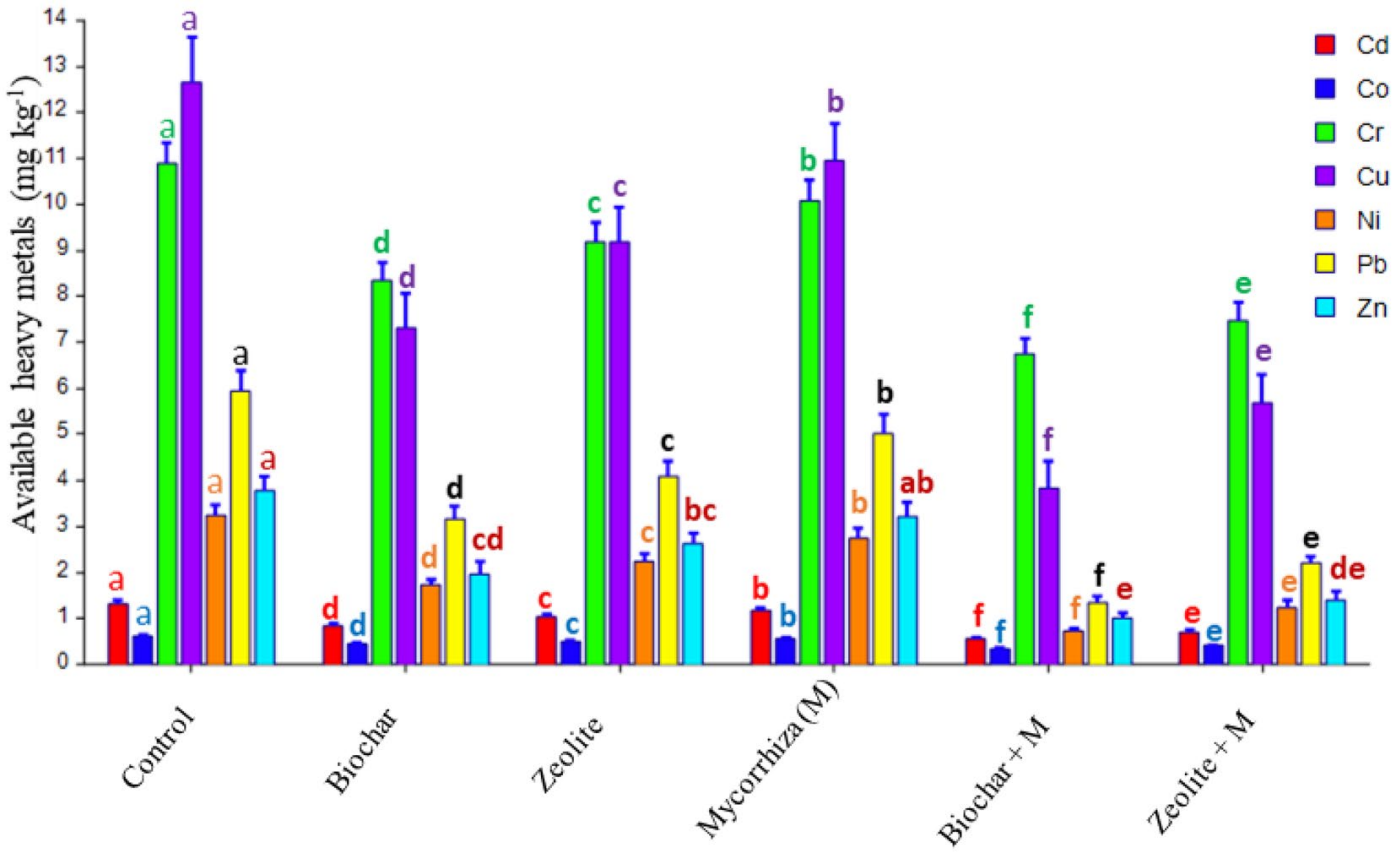
Effect of soil amendments and *mycorrhiza* on the content of available heavy metals in soil (mg kg^**-**1^) after harvest. Error bars are the standard deviation. (Different letters above bars represent significant differences at *P* < 0.05 among various treatments.

The results showed that there were significant effects of different applications on the content of available heavy metals in the soil after harvest (Fig. 2). The content of available heavy metals in the untreated soil (control) varied in the following order: Cu (12.66 mg kg^−1^) > Cr (10.88 mg kg^−1^) > Pb (5.93 mg kg^−1^) > Zn (3.79 mg kg^−1^) > Ni (3.26 mg kg^−1^) > Cd (1.32 mg kg^−1^) > Co (0.62 mg kg^−1^). The values arranged in decreasing order as follows: control > mycorrhiza > zeolite > biochar > zeolite + mycorrhiza > biochar + mycorrhiza. The highest significant reductions over the control were found with biochar + mycorrhiza, which were 59.1%, 44.3%, 38.0%, 69.7%, 77.8%, 77.2% and 73.6% for Cd, Co, Cr, Cu, Ni, Pb and Zn, respectively.

### Soil properties after harvest

**Figure 3:**
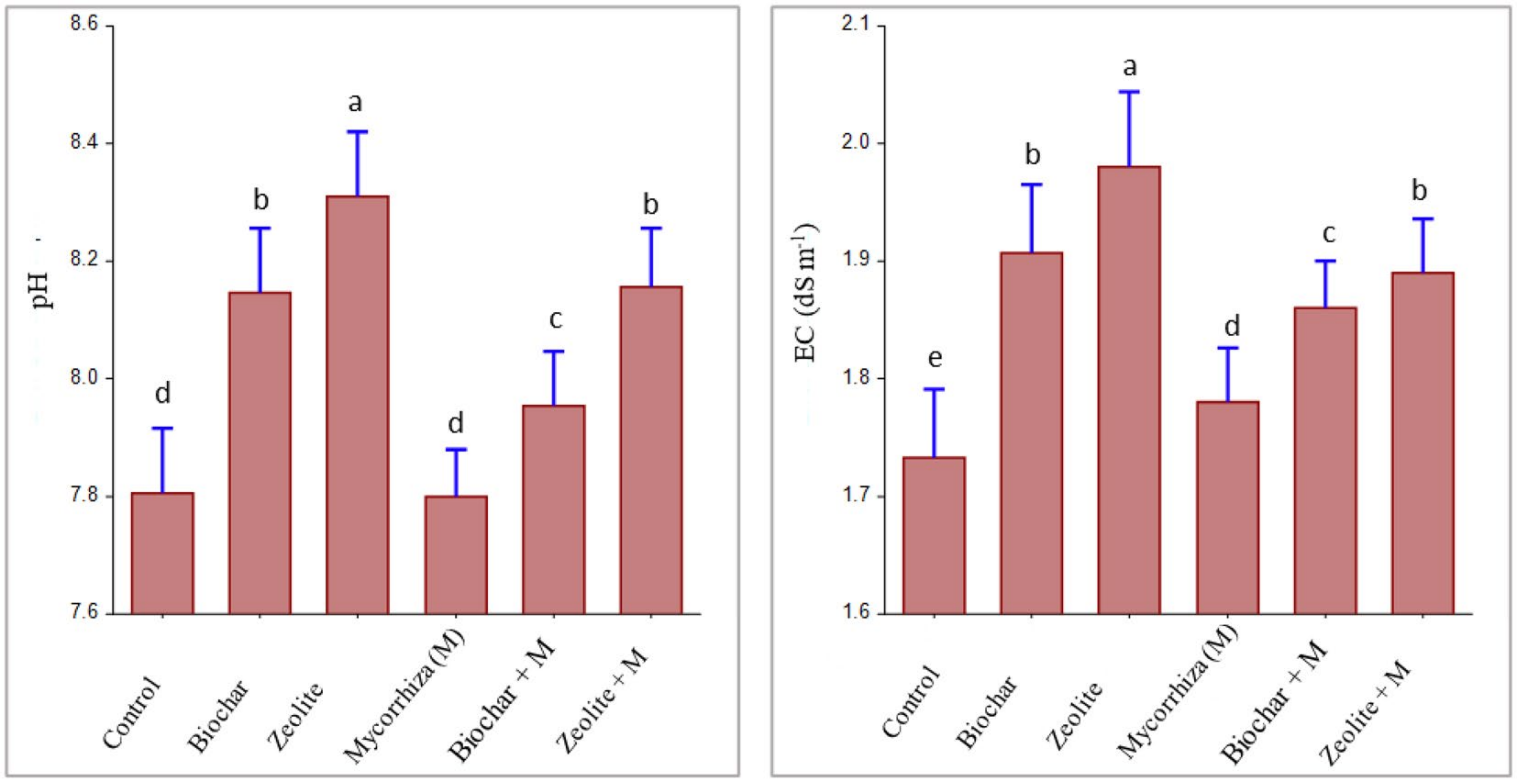
Effect of soil amendments and mycorrhiza on soil pH and EC. Error bars are the standard deviation. (Different letters above bars represent significant differences at *P* < 0.05 among various treatments. Bars with the same letters represent no significant difference).

The application of biochar and zeolite either alone or in combination with mycorrhiza increased significantly soil pH compared to mycorrhiza treatment and untreated soil (Fig. 3), which their soil pH was moderately alkaline (pH = 7.80). All applications increased significantly EC compared to untreated soil. When the biochar and zeolite additions were applied alone, soil pH and EC were significantly increased compared with those observed after mycorrhiza inoculation with them. The highest significant increases over the control were found with zeolite treatment, which were 8.31 and 1.98 dS m^–1^ for soil pH and EC, respectively.

## Discussion

The efficiency of biochar, zeolite, and mycorrhiza in reducing heavy metal bioavailability in soil and bioaccumulation in plants, as well as in promoting the growth of cowpea grown in highly polluted soil, was investigated in the current study. The reduced plant biomass from the control treatment (Fig. 1) must be explained by the excessive concentration of heavy metals in the plant (Table 1) that caused plant toxicity and resulted in a decrease in biomass production^6^. Nawab et al.^18^ found that the root and shoot growth of pea plants decreased with the high toxicity of Cr, Ni, Zn, Pb, and Cd in soil. The present study found that mycorrhiza can boost shoot biomass and adding biochar and zeolite to the soil can promote it (Fig. 1). The stimulating dry biomass by application of biochar and zeolite in combination with mycorrhiza may be attributed to the important role of biochar, zeolite, and mycorrhiza in lowering heavy metal bioavailability (Fig. 2) resulting in lower plant uptake and phytotoxicity of heavy metals, which led to improved plant growth (Fig. 1). This finding was in line with the results of prior studies on biochar^6^, zeolite^19^ and mycorrhiza^20^ when they were used to remediate soil that had been polluted by toxic metals.

Biochar and zeolite boost plant growth during the remediation process by a variety of mechanisms, including toxic metal adsorption, higher soil water retention, encouraging plant growth-promoting microorganisms, and increased root development^19,21^. In our study, zeolite was found to be less effective than biochar as a soil amendment, despite a converse result being found when lower addition rates (0.5%) were used^19^. The increases in dry biomass as a result of biochar and zeolite treatment can be explained by increased nutrient availability and uptake in the soil and/or plant^22,23^ and the improvement in soil physio-chemical properties^3,24^.

Moreover, Bashir et al.^25^ reported that biochar improved nodule formation in mungbean plants, microbial activity in the soil, and increased shoot fresh biomass. Increased mycorrhizal colonization in the soil causes an increase in plant biomass^26^. Mycorrhiza help plants absorb water and nutrients by forming a pervasive hyphal network, increasing the efficiency of chemical fertilizers^27^. In metal-contaminated soils, mycorrhiza has been found in association with plant roots and has been suggested to protect plants against heavy metal toxicity and improve plant development^28^. Plants grown in heavy metal-contaminated soils can benefit from mycorrhiza by facilitating nutrient uptake, protecting them from metal toxicity, sequestering HMs into mycorrhiza structures, and improving phytostabilization ^14,23^ On the other hand, using biochar in combination with other amendments, such as soil microorganisms, can boost biochar's trace metal remediation capacity and improve crop growth^29^. The stimulative effect of biochar and mycorrhiza combined treatment could be attributed to the benefits of biochar to mycorrhiza as a source of reduced carbon compounds and available nutrients ^12,23^.

Biochar amendment increases the physicochemical properties of soils, making them more suitable for mycorrhiza colonization by promoting spore germination, hyphal branching, and mycorrhiza growth ^30,31^. Besides, biochar absorbs the substances that are hazardous to mycorrhizal fungus, effectively lowering the toxicity of many heavy metals^32^. Guo and Li^32^ found that the combination of biochar and mycorrhiza protected the plants from heavy metal toxicity and promoted plant growth and the effect of their combined inoculation were greater than a single one.

The heavy metal concentrations in roots and shoots of the plants grown under biochar or zeolite combined with mycorrhiza inoculation applications were lower than in single applications, which were related to the interaction of biochar or zeolite with mycorrhiza. These significant reductions could be attributed to enhanced immobilization of available heavy metals in soil (Fig. 2), as well as a diluting impact due to increased plant biomass (Fig. 1). Mycorrhiza reduces heavy metal concentrations in roots and shoots, such as Cd, Cu, Mn, and Zn^26^. Mycorrhiza's efficiency to reduce Cd uptake increased after zeolite addition to the soil^15^. The application of biochar with mycorrhiza in heavy metal-contaminated soils reduced significantly heavy metal levels in the shoots by reducing heavy metal translocation from the roots^32^.

The bioaccumulation factor results indicate that all treatments of soil amendments and mycorrhiza inhibited the bioaccumulation of selected heavy metals in cowpea (Table 2). The accumulation of heavy metals in cowpea root and shoot tissues was smaller than the accumulation in soil, as evidenced by the fact that none of the BCF values exceeded one. The lower BCF values were most likely due to biochar and mycorrhiza's higher efficiency in reducing heavy metal uptake (Table 1). In general, adding biochar or mycorrhiza to soil reduced the bioavailable concentration of heavy metals in the soil, resulting in lower root uptake^6,33^. Metals adsorb on the surfaces of biochar and mycorrhiza, reducing their concentrations and mobilization in the soil, as well as their translocation in plants^34,35^. Moreover, biochar increase the pH, organic carbon content, and cation exchange capacity of the soil leading to lower the phytoavailability of heavy metals^36,37^.

The decrease in TF values (Table 3) could be related to metal precipitation in the root tissues as presented in Table 1. In comparison to shoots, a major portion of the heavy metals was contained in the roots. The use of biochar in combination with mycorrhiza is a viable treatment for lowering TF levels. According to the findings of prior studies, the TF values of heavy metals from roots to shoots were significantly reduced after biochar was applied to the multi-metal contaminated soil^6,38^. Moreover, mycorrhiza is effective in reducing heavy metal transport from the root to the shoots^26^, because heavy metals can be retained in mycorrhizal structures^39^. Plant-mycorrhiza symbiosis could enable selective transport mechanisms of both essential minerals and heavy metals^40^. Mixing biochar with mycorrhiza in metal-contaminated soils reduced significantly heavy metal translocation from the roots^23,32^^**.**^

The increase in soil pH and organic matter will promote heavy metal immobilization^41^, resulting in a reduction in the plant's heavy metal uptake^6,42^. Both zeolite and biochar supply alkalinity to the soil causing insoluble metals ^36,43^. In this study, the pH value of soil is increased with the application of biochar and zeolite (Fig. 3), which reduced the content of available heavy metals in the soil after harvest (Fig. 2). The soil organic matter was raised when biochar was applied^44,45^. According to Chen et al.^42^ average concentrations of Cd, Pb, Cu, and Zn in plant tissues are lowered in comparison with control by 38%, 39%, 25%, and 17%, respectively, since biochar has a high pH value and increases organic matter content in the soil. Several studies proved the biochar and zeolite role in reducing the availability of heavy metals in soil^6,36,37,46^. Both biochar and zeolite reduce metal solubility in soils by many mechanisms: (1) supplying alkalinity and consequent pH rise^32,43^ (2) promoting sorption by surface complexation^36,45^, (3) increasing cation exchange retention^19^, (4) organic complexation^19,47^ , and (5) reduction of hydraulic conductivity and pore size of soils^19^. Due to metal binding and complex formation, zeolite was effective in lowering heavy metal mobility^46^. The positive cations in zeolite can exchange with specific heavy metal cations in soil solutions, giving it a high capacity for cation exchange^46,48^. Biochar can absorb soil heavy metals by ion exchange process, complexation, precipitation functions, electrostatic attraction, or transforming metals from an inorganic form to an organic form that altered the pollutants' bioavailability and mobility^7^. On the other hand, mycorrhizal soil inoculation could be vital in immobilizing heavy metals in the soil and altering their availability to plants^13,27,28^.

Mycorrhiza acts as a physical barrier by sequestering the heavy metals into mycorrhizal structures and immobilizing heavy metals in plant roots^28^. On the surface of the fungal structure, positive charge particles such as amino acids, cysteine, glutathione and thiol groups adsorb and later reduce heavy metals from one form to another^35^. Also, mycorrhiza creates extracellular polymeric substances, which have carboxyl, phosphoric, amine and hydroxyl functional groups and have a high affinity for metal adsorption through chelation, surface precipitation, and ion exchange mechanisms^49^. The fungal structure is often much finer than roots, and it has a strong potential to chelate heavy metals and reduce their bioavailability in the rhizosphere through metal speciation^50,51^. Mycorrhiza decreases heavy metal toxicity in plants by keeping heavy metals in mycorrhizal structures such as the fungal mycelium and vesicles, where high quantities of heavy metals are concentrated, preventing their mobilization to aerial plant tissues^27^. Moreover, many studies have shown that combining mycorrhiza and biochar has a positive impact on lowering bioavailable heavy metals in soil^14,30,52^.

The increase in soil pH caused by biochar and zeolite applications is due to their high pH (Table 4). Alkalinity is supplied to the soil by both zeolite and biochar^43^. The increase in soil pH after the application of biochar alone or in combination with mycorrhiza may be related to the pH of biochar itself (Fig. 3). The increase in soil pH is due to the dissolution of carbonates and hydroxides mainly present in the applied biochar's ash friction^7,53^. In this respect, Guo and Li^32^ found that mycorrhiza has no effect on soil pH. Zhuo et al.^30^ found that soil treated with a combination of biochar and mycorrhiza had a considerable increase in pH. Moreover, various researchers reported that biochar and zeolite increase the soil EC value^54^.

**Table 4:** Selected properties of studied biochar and zeolite.

Properties	Zeolite	Biochar
Cation exchange capacity CEC (cmol kg^-1^)	155	28
pH	8.67	9.43
EC (dS m^–1^) (1:10)	2.03	1.07
Total C (%)	8.9	77.1
DTPA-extractable heavy metals, mg kg^-1^		
Cd (mg kg^−1^)	0.022	0.012
Co (mg kg^−1^)	0.147	2.54
Cr (mg kg^−1^)	0.123	9.86
Cu (mg kg^−1^)	1.710	2.94
Ni (mg kg^−1^)	0.162	2.81
Pb (mg kg^−1^)	9.348	1.06
Zn (mg kg^−1^)	5.231	4.16

## Conclusions

Heavy metal pollution of agricultural soil causes severe threats to ecosystem sustainability and human health, and it has become a major serious concern. The development of suitable control and remediation strategies for heavy metal-contaminated soil has become critical. The results of the present study suggest that the combined application of biochar or zeolite with mycorrhiza in metal-contaminated soil can improve cowpea growth, decrease metal bioavailability and mobility, and reduce metal uptake. Biochar was shown to be more effective than zeolite. This avenue of study will help in the remediation of metal-contaminated soils, resulting in improved plant growth and lower bioconcentration and translocation factors in a cost-effective and environmental technique. More specialized field-scale experiments are necessary to evaluate the potential role of biochar and mycorrhiza in the remediation of heavy metal-contaminated soils and validate their practical application with a variety of plant and climate conditions.

## Materials and methods

### Soil, zeolite, biochar and mycorrhiza

The soil used in this study was collected from an agricultural field near Mansoura, Dakahlia Governorate, Egypt (31°25′16.1′′E, 31°03′07.8′′N), at a depth of 20 cm. A standard approach narrated in Ryan et al.^55^was used to examine the physio-chemical properties of the studied soil. The properties of this soil are presented in Table 5.

**Table 5:** Selected soil physical–chemical properties of the soil before sowing cowpea seeds.

Parameter	Value
Clay (%)	39.66
Silt (%)	37.71
Sand (%)	23.63
Texture	Clay-loam
Organic matter (%)	1.74
EC (dS m^–1^) (1:10)	2.18
pH	7.93
Total Cd (mg kg^−1^)	2.36
Total Co (mg kg^−1^)	15.14
Total Cr (mg kg^−1^)	113.72
Total Cu (mg kg^−1^)	16.58
Total Ni (mg kg^−1^)	22.85
Total Pb (mg kg^−1^)	29.32
Total Zn (mg kg^−1^)	91.57

The zeolite clinoptilolite which a hydrated aluminosilicate mineral that contain alkali and alkaline earth metals (Na, K, Ca, and Mg) was used in this experiment. It was purchased from a company in Giza, Egypt. The zeolite was sieved at 0.2 mm before being used. The biochar was produced using dried woods taken from mature trees of mango as feedstocks. They were exposed to direct sunshine before being sliced into little 10 cm pieces. Dry wood chips were covered thoroughly with aluminum foil to provide an oxygen-limited condition and pyrolyzed at 500 °C for 5 h in muffle furnace (Magma Therm, MT-1200–10-B2, Turkey). Biochar was cooled to room temperature before being crushed using a stainless-steel mill and sieved with a 2 mm mesh size. Mycorrhiza fungi used in this experiment were obtained from Soil, Water and Environment Research Institute, ARC, Egypt. The mycorrhiza used is an endomycorrhizal biofertilizer with 250 spores in 1 g of the carrier. The properties of zeolite and biochar used in this study are shown in Table 4.

### Pot experiment

The outdoor pot experiment was conducted at Mansoura Horticulture Research Station, Dakahlia Governorate, Egypt, during the summer season of 2021. The experiment was conducted with a completely randomized design in four replications. The following six treatments were applied: zeolite, biochar, mycorrhiza, mixtures of zeolite and mycorrhiza, mixtures of biochar and mycorrhiza, and control (soil without any modifications). Both biochar and zeolite were applied at a rate of 2% w/w and completely mixed with soil alone to obtain homogeneity.

To prepare the artificially polluted soil, all of the pots were contaminated with a solution of seven heavy metal salts (CdSO_4_.8H_2_O, CoCl_2_.6H_2_O, Cr_2_(SO_4_)_3_, CuSO_4_5H_2_O, Ni-SO_4_6H_2_O, Pb(C_2_H_3_O_2_)_2_ and ZnSO_4_7H_2_O) at a concentration of 3.2, 10, 85, 50, 50, 70 and 130 mg kg^-1^ for Cd, Co, Cr, Cu, Ni, Pb and Zn, respectively. The water solution with toxic metals was thoroughly mixed into the soil to ensure homogeneity then the plastic pots with a capacity of 7 kg were filled with contaminated soil. The pots were left to equilibrate for three weeks before seed sowing. During this time, the moisture content of the soil was maintained at 65 percent of its field capacity.

After that, on 6 June 2021, soil was inoculated with mycorrhiza. To this end, the mycorrhizal biofertilizer at the rate of 1.5 g soil kg^-1^ was placed in the pots at a depth of 3 cm from the soil surface. Then, ten seeds of cowpea cv. Cream 7 were sown per pot at 2 cm deep and irrigated with tap water. After a week of emergence, the seedlings were trimmed to four of uniform size in each pot. The water content was kept at the field capacity level. All pots were irrigated with NPK fertilizer solution at a level of 60–45-45 kg per hectare, respectively, after 7 days of sowing.

The roots and shoots of plants in each pot were picked separately after five weeks of growth and cleaned thoroughly with tap water followed by distilled water to eliminate dust and soil, and then their fresh weights were recorded. After drying them for 72 h at 70 °C, the dry weights were recorded, and the samples were ground into a fine powder and placed in paper bags for heavy metal analysis. After plant harvests, a soil sample was taken from each pot, air-dried, crushed, sieved through a 2 mm sieve, and stored for physical and chemical analysis.

### Analysis and measurements

For particle-size analysis, the hydrometer method was used. Jackson's method^56^ was used to assess soil pH, organic matter (OM) content, and electrical conductivity (EC). The elemental content of C (carbon) was determined using the Thermo Scientific Flash 2000 elemental analyzer. The total and available concentrations of Cd, Co, Cr, Cu, Ni, Pb and Zn in the extracts were determined using Thermo Scientific TM ICAPTM 7000 Plus Series ICP-OES Ammann^57^. The available heavy metals were extracted by 0.005 M DTPA at pH 7 according to Lindsay and Norvel^58^.

The bioconcentration factor (BCF) and translocation factor (TF) of each heavy metal were determined according to Padmavathiamma and Li^59^, to evaluate the heavy metal absorption and translocation. The ratio of metal concentration in plant tissues to metal concentration in the soil is known as BCF, while the ratio of metal concentration in the plant shoot to metal concentration in the plant root is known as TF.

### Statistical analysis

The ANOVA technique was used to calculate statistical analyses by Costat computer software according to Snedecor and Cochran^60^. The Least Significant Difference (LSD) test was used to evaluate the differences between means. The significance of the difference was determined using the P = 0.05 value.

### Research involving plants statement

This study was developed with commercial seeds, therefore nonexotic or at risk of extinction, under controlled conditions, meeting all institutional, national and international guidelines and legislation for cultivated plants.

## Data Availability

All data generated and/ or analyzed during this study are available from the corresponding author on reasonable request.

## References

[1] Woodford, C. Land pollution. Retrieved from https://www.explainthatstuff.com/land-pollution.html (2013).

[2] Hu JL, Wu SC, Wu FY, Leung HM, Lin XG, Wong MH (2013). Arbuscular mycorrhizal fungi enhance both absorption and stabilization of Cd by Alfred stonecrop (Sedum alfredii Hance) and perennial ryegrass (*Lolium perenne* L.) in a Cd contaminated acidic soil. Chemosphere.

[3] Bashir S, Salam A, Rehman M, Khan S, Gulshan AB, Iqbal J, Shaaban M, Mehmood S, Zahra A, Hu H (2019). Effective role of biochar, zeolite, and steel slag on leaching behavior of Cd and its fractionations in soil column study. Bull Environ. Contam. Toxicol..

[4] He L, Zhong H, Liu G, Dai Z, Brookes PC, Xu J (2019). Remediation of heavy metal contaminated soils by biochar: Mechanisms, potential risks and applications in China. Environ. Pollut..

[5] Li H, Dong X, da Silva EB, de Oliveira LM, Chena Y, Ma LQ (2017). Mechanisms of metal sorption by biochars: biochar characteristics and modifications. Chemosphere.

[6] Ibrahim EA, El-Sherbini MAA, Selim EM (2022). Effects of biochar on soil properties, heavy metal availability and uptake, and growth of summer squash grown in metal-contaminated soil. Sci. Hortic..

[7] Jatav HS, Rajput VD, Minkina T, Singh SK, Chejara S, Gorovtsov A, Barakhov A, Bauer T, Sushkova S, Mandzhieva S, Burachevskaya M, Kalinitchenko VP (2021). Sustainable approach and safe use of biochar and its possible consequences. Sustainability.

[8] Khan TF, Didar-Ul-Alam M (2018). Effects of biochar on legume-Rhizobium symbiosis in soil. Bangladesh J. Bot..

[9] Akbar S, Khatoon S, Shehnaz R, Hussain T (1999). Natural zeolites: Structures, classification, origin, occurrence and importance. Sci. Int. (Lahore).

[10] Terzano R, Spagnuolo M, Medici L, Tateo F, Ruggiero P (2005). Zeolite synthesis from pre-treated coal fly ash in presence of soil as a tool for soil remediation. Appl. Clay Sci..

[11] Lahori AH, Mierzwa-Hersztek M, Demiraj E, Sajjad RU, Ali I, Shehnaz H, Aziz A, Zuberi MH, Pirzada AM, Hassan K, Zhang Z (2020). Direct and residual impacts of zeolite on the remediation of harmful elements in multiple contaminated soils using cabbage in rotation with corn. Chemosphere.

[12] Barna G, Makó A, Takács T, Skic K, Füzy A, Horel Á (2020). Biochar alters soil physical characteristics, arbuscular mycorrhizal fungi colonization, and glomalin production. Agronomy.

[13] Davamani, V., Lourduraj, A. C. & Velmurugan, M. Influence of VAM in bioremediation of environmental pollutants. In: *Mycorrhizal Biotechnology* (eds Thangadurai, D., Busso, C. A., Hijri M.). 100–115 (Science Publishers, 2010).

[14] Riaz M, Kamran M, Fang Y, Wang Q, Cao H, Yang G, Deng L, Wang Y, Zhou Y, Anastopoulos I, Wang X (2021). Arbuscular mycorrhizal fungi-induced mitigation of heavy metal phytotoxicity in metal contaminated soils: A critical review. J. Hazard Mater..

[15] Baghaie AH, Aghili F, Jafarinia R (2019). Soil-indigenous arbuscular mycorrhizal fungi and zeolite addition to soil synergistically increase grain yield and reduce cadmium uptake of bread wheat (through improved nitrogen and phosphorus nutrition and immobilization of Cd in roots). Environ. Sci. Pollut. Res. Int..

[16] Ailenokhuoria BV, Omolekan TO (2019). Heavy metal, proximate and antioxidant composition of five cultivars of cowpea (*Vigna unguiculata*) in Ibadan, Oyo State Nigeria. J. Food Sci. Nutr. Res..

[17] Mehboob S, Iqbal MZ, Shafiq M, Kabir M, Farooqi Z (2018). Effects of Lead on different seedling growth attributes of cowpea (*Vigna unguiculata* L.). Asian J. Res. Crop Sci..

[18] Nawab J, Ghani J, Khan S, Xiaoping W (2018). Minimizing the risk to human health due to the ingestion of arsenic and toxic metals in vegetables by the application of biochar, farmyard manure and peat moss. J. Environ. Manag..

[19] Głąb T, Gondek K, Mierzwa-Hersztek M (2021). Biological effects of biochar and zeolite used for remediation of soil contaminated with toxic heavy metals. Sci. Rep..

[20] Cui G, Ai S, Chen K, Wang X (2019). Arbuscular mycorrhiza augments cadmium tolerance in soybean by altering accumulation and partitioning of nutrient elements, and related gene expression. Ecotoxicol Environ. Saf..

[21] El-Naggar A, Lee M-H, Hur J, Lee YH, Igalavithana AD, Shaheen SM, Ryu C, Rinklebe J, Tsang DCW, Ok YS (2020). Biochar-induced metal immobilization and soil biogeochemical process: an integrated mechanistic approach. Sci. Total Environ..

[22] Bashir S, Hussain Q, Shaaban M, Hu H (2018). Efficiency and surface characterization of different plant derived biochar for cadmium (Cd) mobility, bioaccessibility and bioavailability to Chinese cabbage in highly contaminated soil. Chemosphere.

[23] Zhang F, Liu M, Li Y, Che Y, Xiao Y (2019). Effects of arbuscular mycorrhizal fungi, biochar and cadmium on the yield and element uptake of *Medicago sativa*. Sci. Total Environ..

[24] Cataldo E, Salvi L, Paoli F, Fucile M, Masciandaro G, Manzi D, Masini CM, Mattii GB (2021). Application of zeolites in agriculture and other potential uses: A review. Agronomy.

[25] Bashir S, Hussain Q, Akmal M, Riaz M, Hu H, Ijaz SS, Iqbal M, Abro S, Mehmood S, Ahmad M (2017). Sugarcane bagasse-derived biochar reduces the cadmium and chromium bioavailability to mash bean and enhances the microbial activity in contaminated soil. J. Soils Sediment.

[26] Atakan A, Özkaya HÖ, Erdoğan O (2018). Effects of arbuscular mycorrhizal fungi (AMF) on heavy metal and salt stress. Turk. J. Agric. Food Sci. Technol..

[27] Dhalaria R, Kumar D, Kumar H, Nepovimova E, Kuča K, Torequl IM, Verma R (2020). Arbuscular mycorrhizal fungi as potential agents in ameliorating heavy metal stress in plants. Agronomy.

[28] Ma Y, Rajkumar M, Oliveira RS, Zhang C, Freitas H (2019). Potential of plant beneficial bacteria and arbuscular mycorrhizal fungi in phytoremediation of metal contaminated saline soils. J. Hazard. Mater..

[29] Haider FU, Wang X, Farooq M, Hussain S, Cheema SA, Ain NU, Virk AL, Ejaz M, Janyshova U, Liqun C (2022). Biochar application for the remediation of trace metals in contaminated soils: Implications for stress tolerance and crop production. Ecotoxicol. Environ. Saf..

[30] Zhuo F, Zhang X, Lei L, Yan T, Lu R, Hu Z, Jing Y (2020). The effect of arbuscular mycorrhizal fungi and biochar on the growth and Cd/Pb accumulation in Zea mays. Int. J. Phytoremediation.

[31] Ndiate NI, Qun CL, Nkoh JN (2022). Importance of soil amendments with biochar and/or arbuscular mycorrhizal fungi to mitigate aluminum toxicity in tamarind (*Tamarindus indica* L.) on an acidic soil: A greenhouse study. Heliyon.

[32] Guo M, Li H (2019). Effects of iron-modified biochar and AMF inoculation on the growth and heavy metal uptake of *Senna occidentalis* in heavy metal-contaminated soil. Pol. J. Environ. Stud..

[33] Ali A, Guo D, Zhang Y, Sun X, Jiang S, Guo Z, Huang H, Liang W, Li R, Zhang Z (2017). Using bamboo biochar with compost for the stabilization and phytotoxicity reduction of heavy metals in mine-contaminated soils of China. Sci. Rep..

[34] Lucchini P, Quilliam RS, DeLuca TH, Vamerali T, Jones DL (2014). Increased bio-availability of metals in two contrasting agricultural soils treated with waste wood-derived biochar and ash. Environ. Sci. Pollut. Res..

[35] Joutey NT, Sayel H, Bahafid W, El Ghachtouli N (2015). Mechanisms of hexavalent chromium resistance and removal by microorganisms. Rev. Environ. Contam. Toxicol..

[36] Alkharabsheh HM, Seleiman MF, Battaglia ML, Shami A, Jalal RS, Alhammad BA, Almutairi KF, Al-Saif AM (2021). Biochar and its broad impacts in soil quality and fertility, nutrient leaching and crop productivity: A review. Agronomy.

[37] Antonangelo, J. A. & Zhang, H. The use of biochar as a soil amendment to reduce potentially toxic metals (PTMs) phytoavailability. In: *Applications of Biochar for Environmental Safety* (eds Abdelhafez, A. A., Abbas, M. H. H.) *Intech Open* (2020). 10.5772/intechopen.92611

[38] Antonangelo J, Zhang H (2021). Influence of biochar derived nitrogen on cadmium removal by ryegrass in a contaminated soil. Environments.

[39] Malcová R, Vosátka M, Gryndler M (2003). Effects of inoculation with Glomus intraradices on lead uptake by *Zea mays* L. and *Agrostis capillaris* L.. Appl. Soil Ecol..

[40] Begum N, Qin C, Ahanger MA, Raza S, Khan MI, Ashraf M, Ahmed N, Zhang L (2019). Role of arbuscular mycorrhizal fungi in plant growth regulation: implications in abiotic stress tolerance. Front. Plant Sci..

[41] He Y, Lin H, Jin X, Dong Y, Luo M (2020). Simultaneous reduction of arsenic and cadmium bioavailability in agriculture soil and their accumulation in *Brassica chinensis* L. by using minerals. Ecotoxicol. Environ. Saf..

[42] Chen G, Shah KJ, Shi L, Chiang PC, You Z (2019). Red soil amelioration and heavy metal immobilization by a multi-element mineral amendment: Performance and mechanisms. Environ. Pollut..

[43] Zheng X, Chen M, Wang J, Liu Y, Liao Y, Liu Y (2020). Assessment of zeolite, biochar, and their combination for stabilization of multi metal-contaminated soil. ACS Omega.

[44] El-Naggar A, Lee SS, Awad YM, Yang X, Ryu C, Rizwan M, Rinklebe J, Tsang DCW, Ok YS (2018). Influence of soil properties and feedstocks on biochar potential for carbon mineralization and improvement of infertile soils. Geoderma.

[45] Yang CD, Lu SG (2020). Dynamic effects of direct returning of straw and corresponding biochar on acidity, nutrients, and exchangeable properties of red soil. Huan Jing Xue Huanjing Kexue.

[46] Moeen M, Qi T, Hussain Z, Ge Q, Maqbool Z, Jianjie X, Kaiqing F (2020). Use of zeolite to reduce the bioavailability of heavy metals in a contaminated soil. J Ecol Eng.

[47] Yang X, Igalavithana AD, Oh SE, Nam H, Zhang M, Wang CH, Kwon EE, Tsang DCW, Ok YS (2018). Characterization of bioenergy biochar and its utilization for metal/metalloid immobilization in contaminated soil. Sci. Total Environ..

[48] Budianta, W. Laboratory study on the use of natural zeolite from Gunungkidul, Indonesia for Cu, Pb, Zn and Cd immobilization in soil. In *E3S Web of Conferences*, vol. **200**, 06006 (2020). 10.1051/e3sconf/202020006006.

[49] Li WW, Yu HQ (2014). Insight into the roles of microbial extracellular polymer substances in metal biosorption. Bioresour. Technol..

[50] Malekzadeh E, Aliasgharzad N, Majidi J, Abdolalizadeh J, Aghebati-Maleki L (2016). Contribution of glomalin to Pb sequestration by arbuscular mycorrhizal fungus in a sand culture system with clover plant. Eur. J. Soil Biol..

[51] Wang W, Shi J, Xie Q, Jiang Y, Yu N, Wang E (2017). Nutrient exchange and regulation in arbuscular mycorrhizal symbiosis. Mol. Plant..

[52] Khan MA, Ramzani PMA, Zubair M, Rasool B, Khan MK, Ahmed A, Khan SA, Turan V, Iqbal M (2020). Associative effects of lignin derived biochar and arbuscular mycorrhizal fungi applied to soil polluted from Pb acid batteries effluents on barley grain safety. Sci. Total. Environ..

[53] da Silva ICB, Basilo JJN, Fernandes LA, Colen F, Sampaio RA, Frazáo LA (2017). Biochar from different residues on soil properties and common bean production. Sci. Agric..

[54] Szatanik-Kloc A, Szerement J, Adamczuk A, Józefaciuk G (2021). Effect of low zeolite doses on plants and soil physicochemical properties. Materials.

[55] Ryan, J., Estefan, G. & Rashid, A. Soil and plant analysis laboratory manual. In *International Center for Agricultural Research in the Dry Areas (ICARDA)*, *Islamabad, Pakistan* 172 (2001).

[56] Jackson ML (1973). Soil Chemical Analysis.

[57] Ammann AA (2007). Inductively coupled plasma mass spectrometry (ICP MS): a versatile tool. J. Mass Spectrom..

[58] Lindsay WL, Norvel WA (1978). Development of a DTPA soil test for zinc, iron, manganese and copper. Soil Sci. Soc. Am. J..

[59] Padmavathiamma PK, Li LY (2007). Phytoremediation technology: Hyper-accumulation metals in plants. Water Air Soil Pollut..

[60] Snedecor, G. W., Cochran, W. G. *Statistical Methods* 8th Edn., 503 (Iowa State University Press, Iowa, 1989).

